# Enhanced accumulation of anticancer compounds in *C. roseus* hairy root cultures through elicitation and precursor feeding

**DOI:** 10.1038/s41598-026-36185-y

**Published:** 2026-02-03

**Authors:** Mohamed R. Rady, Dalia M. Mabrouk, Alaa M. El-Minisy, Mona M. Ibrahim

**Affiliations:** 1 Department of Plant Biotechnology, Biotechnology Research Institute, National Research Centre, 12622 Cairo, Egypt; 2 Department of Cell Biology, Biotechnology Research Institute, National Research Centre, 12622 Cairo, Egypt

**Keywords:** *Catharanthus roseus*, Hairy root cultures, HPLC, Gene expression, Biotechnology, Cancer, Drug discovery, Molecular biology, Plant sciences

## Abstract

*Catharanthus roseus* is a medicinal plant known for producing numerous indole terpenoid alkaloids. This study investigated the effects of methyl jasmonate, yeast extract, tryptophan, and tryptamine on the accumulation of four key alkaloids—ajmalicine, catharanthine, vincristine, and vinblastine—in hairy root cultures of *C. roseus*. Additionally, the expression levels of two biosynthetic genes, *tryptophan decarboxylase (TDC)* and *strictosidine synthase (STR)* were analyzed to explore potential transcriptional responses to elicitation. All concentrations of methyl jasmonate (MeJA) increased the levels of ajmalicine and catharanthine, while MeJA (10 and 250 µM) increased the levels of vinblastine. In contrast, yeast extract (YE) generally suppressed all indole alkaloid production. Tryptophan (TRPh) (50 mg/l) enhanced the production of catharanthine and vinblastine, while tryptamine (TRM) (100 mg/l) enhanced the production of vinblastine. Gene expression analysis revealed that methyl jasmonate (10 and 100 µM), tryptophan (50 and 250 mg/l), and tryptamine (100 mg/l) upregulated TDC and STR expression, whereas yeast extract downregulated these genes. These findings demonstrate that elicitor and precursor treatments can modulate both metabolic output and transcriptional activity in *C. roseus* hairy roots, providing useful insights for optimizing the in vitro production of anticancer compounds.

## Introduction

*Catharanthus roseus* contains important chemicals called vincristine and vinblastine, which are used in cancer treatment. *Catharanthus roseus* contains important chemicals called vincristine and vinblastine, which are used in cancer treatment. Medicinal plants are often referred to as “chemical factories” due to their ability to produce bioactive substances with industrial and pharmaceutical significance. However, a major issue with extracting phytochemicals from regular plant parts is that their growth and production can be significantly influenced by the environment, resulting in low yields of these important medicine-making alkaloids and high market costs. Interest in exploring alternative methods of production for *C. roseus* has grown, as mentioned in Verma et al.‘s study^1^.

Most Terpenoid Indole Alkaloids (TIAs), especially dimeric and bisindole alkaloids are found in small amounts, requiring large quantities of raw materials for compound isolation. For instance, obtaining 1 gram of vinblastine necessitates around 500 kg of plant material. Van der Heijden et al.^2^ proposed an alternative method. Kumar et al.^3^ reported in 2022 that market prices for vinblastine and vincristine supply are estimated at $2 million and $15 million per kilogram, respectively. The limited supply of these drugs is due to insufficient availability of the plants required for their production worldwide. Additionally, the amounts of TIAs produced by regular plants are low, and synthesizing them in large quantities through chemical means is impractical due to high costs and complex structures^4^.Therefore, further research on *C. roseus* will focus on exploiting the plant’s herbal value.

For these reasons, a biotechnological approach using plant biotechnology techniques is being explored as an alternative production method for valuable bioactive metabolites from plants.

Hairy root cultures are currently receiving increased attention as biological matrices for producing valuable metabolites. This is due to their attractive features, such as high genetic stability and relatively fast growth rates. Over the past two decades, significant attention has been directed towards synthesizing these valuable compounds found in hairy roots. The use of hairy root cultures from *C. roseus* has significantly increased the production and stability of secondary metabolites. Many authors have studied the production of secondary metabolites from hairy root cultures of *C. roseus *^5,6^.

Elicitation is a widely used method in biotechnology to enhance the production of secondary metabolites. Elicitors are compounds that activate plant defense mechanisms, promoting the production of secondary metabolites to protect the cells and the plant as a whole^7^. In a study by Rady et al.^8^, the impact of methyl jasmonate and UV-B treatment on the production of anticancer compounds in *C. roseus* was investigated. Mona et al. Researchers examined how light and methyl jasmonate influence the accumulation of cancer-fighting compounds in cell cultures of *C. roseus*.

Vu et al.^9^ studied the in vitro growth and content of vincristine and vinblastine in C. roseus L. hairy roots following elicitors and precursors. An earlier study^10^ indicated that reduced light affects both primary and secondary metabolites and may modify membrane stability in *C. roseus.* Genotypic differences also affected certain metabolic processes. However, recently, Rady et al.^11^ studied the effect of different carbon sources and their concentrations on alkaloid accumulation in transformed root cultures of *C. roseus*.

Scientists have been working on increasing the flow through this pathway by employing various methods, such as introducing genes that encode specific metabolic enzymes into the plant. In one instance, two genes, Tdc and Str, responsible for producing enzymes known as tryptophan decarboxylase (TDC) and strictosidine synthase (STR), were discovered to be regulated by their gene expression in *C. roseus*^12^. *STR* combines tryptamine and the iridoid secologanan to produce strictosidine, which serves as the common starting material for all TIAs. Meanwhile, *TDC* plays a crucial role in linking the initial and subsequent stages of metabolism by converting tryptophan into tryptamine.

Different researchers have studied the effect of elicitors on gene expression levels in treated *C. roseus* hairy roots^13,14^ However, the impact of light on the expression levels of two important genes, *CrTDC* and *CrSTR*, was examined in the leaves of *C. roseus* pooled from different genotypes, as reported by Gholizadeh and colleagues^10^.

The novelty of this study lies in the simultaneous comparison of four elicitors/precursors methyl jasmonate, yeast extract, tryptophan, and tryptamine on both metabolite profiles (ajmalicine, catharanthine, vincristine, and vinblastine) and gene expression (*TDC*, *STR*) in the same hairy root line under identical culture conditions. This integrated approach provides a comprehensive view of the terpenoid indole alkaloid pathway response to elicitation.

The aim of this study was to evaluate the effects of different concentrations of methyl MeJA, yeast extract, tryptophan, and tryptamine on the production of vinblastine, vincristine, catharanthine, and ajmalicine in *C. roseus* hairy root cultures. In addition, the expression levels of the *TDC* and *STR* genes were analyzed to gain insights into the transcriptional regulation of terpenoid indole alkaloid (TIA) biosynthesis.

## Experimental

### Hairy root induction and culture condition

**Fig. 1:**
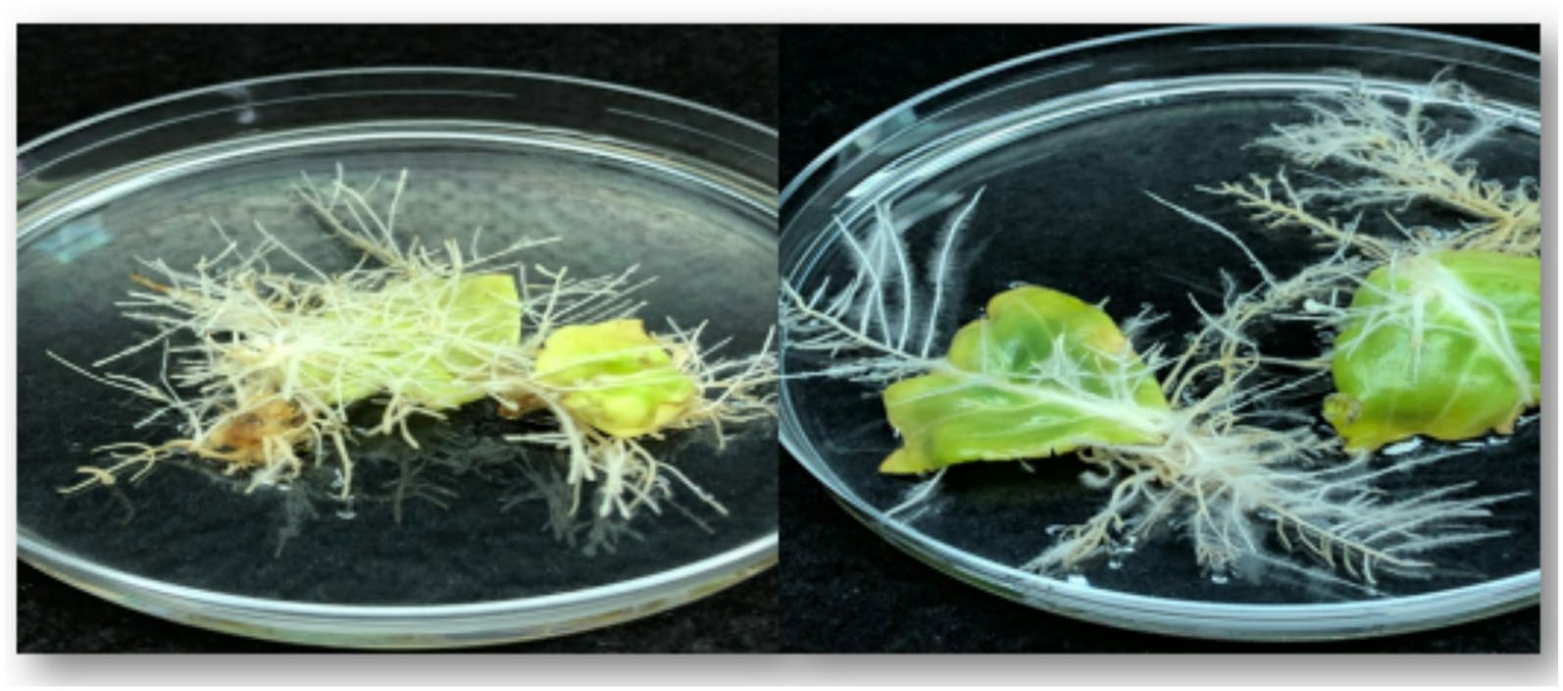
Initiation of *C. roseus* hairy roots from leaf explants after one month.

**Fig. 2:**
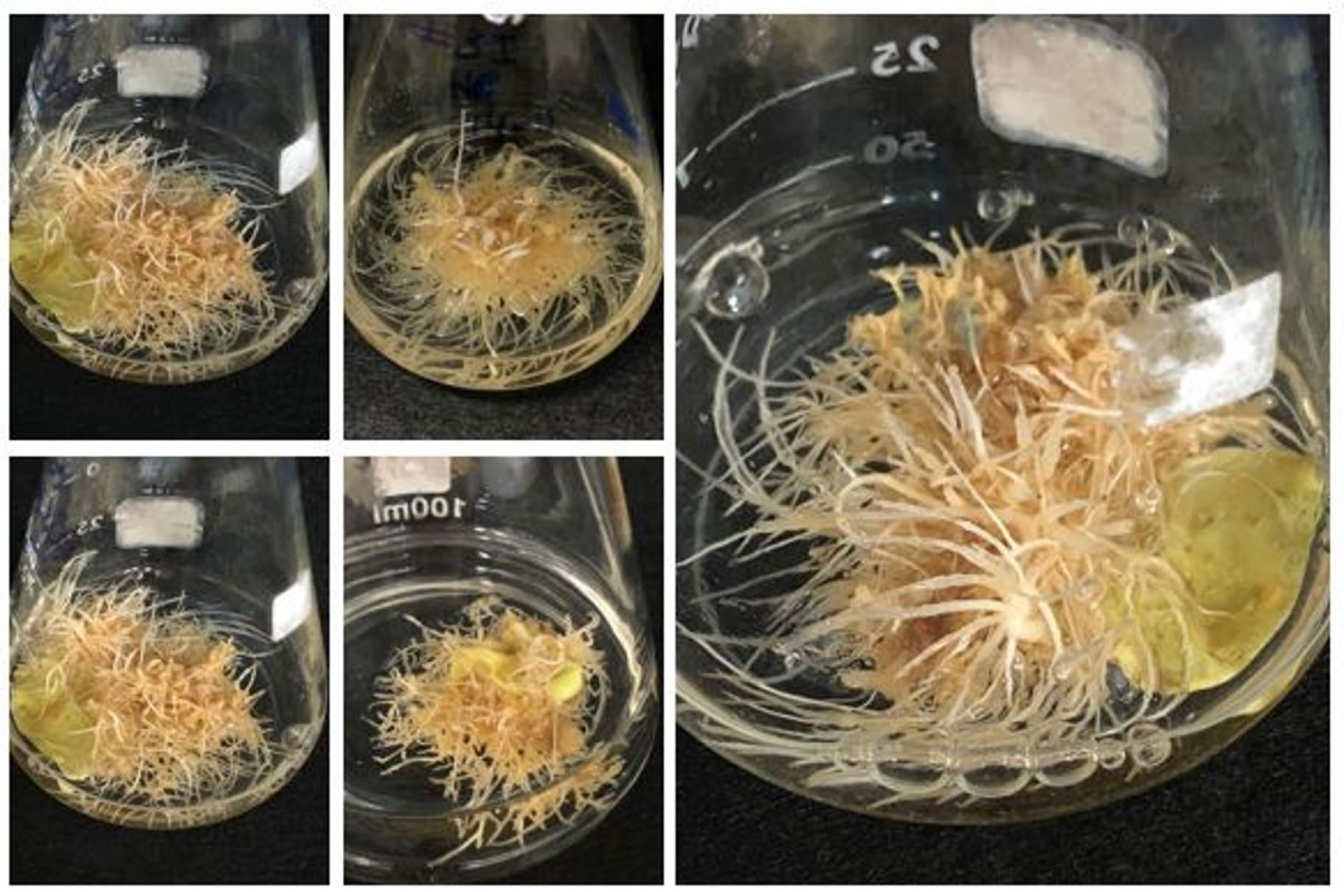
Establishment of hairy root cultures in liquid media.

The hairy root line used in this study was previously generated (Fig. 1) by infecting *C. roseus* leaves with *Agrobacterium rhizogenes* strain ATCC 15,834^11^. The hairy root line was confirmed via PCR for the presence of *rolB* and *rolC* genes^11^. Hairy root cultures were transferred to fresh liquid medium and maintained on 1/2 MS basal liquid medium (Fig. 2). Subcultures were performed every 2 weeks. After several subcultures, the hairy roots were subjected to elicitation treatments.

### Elicitors and precursors feedings

We assessed the effects of different concentrations of methyl jasmonate (0.0, 10, 100, and 250 µM), yeast extract (0.0, 0.5, 1.5, and 2.0 g/l), tryptophan (0.0, 50, 150, and 250 mg/l), and tryptamine (0.0, 100, 150, and 200 mg/l) on hairy root cultures. The concentration ranges were selected based on previous studies in *C. roseus* and related systems: methyl jasmonate (10–250 µM)^15^, yeast extract (0.5–2.0 g/L)^16^, tryptophan (50–250 mg/L)^17^, and tryptamine (100–200 mg/L))^17^. The cultures underwent three subculture stages in liquid medium before being transferred to new liquid medium with the specified supplements.

The cultures were maintained in 50 ml of liquid medium in 250 ml flasks, agitated at 110 rpm using a rotary shaker and kept at 25 °C under an 18-hour photoperiod. After a week of cultivation, the hairy roots were removed from the culture media, freeze-dried, and stored at −20 °C for HPLC analysis. The one-week time point was selected based on preliminary experiments and literature reporting alkaloid induction within this period^18^. The control group consisted of hairy roots cultured in elicitor- and precursor-free ½ MS liquid medium.

### High performance liquid chromatography (HPLC) analysis

A sample of approximately 0.5 g was placed into a 10-ml centrifuge tube and submerged in 5 milliliters of an extracting solution. This solution consisted of a 2% formic acid mixture in a 50:50 volume ratio of water and methanol. The sample was left to soak of 2 h. After soaking, the mixture was **s**onicated for 30 min and then centrifuged in a machine for 10 min at a speed of 4000 revolutions per minute.

According to Hanafy et al. (2016), the analysis was performed using an Agilent 1260 Infinity HPLC series (Agilent, USA) with a quaternary pump. The column used was a Kinetic 5 mm EVO C18 100 mm x 4.6 mm (Phenomenex, USA), operated at a temperature of 30 °C. The sample was then transferred to a separate tube, filtered using a 0.2 μm PTFE syringe filter, and subsequently injected into the HPLC system. The separation process was carried out using a ternary linear isocratic elution with (A) HPLC grade water containing 2% formic acid (v/v) and (B) methanol. The injected volume was 20 ml. Detection was performed using a WWD detector set at 254 nm. A limitation of the current study is pooling three samples per treatment for a single, non-repeated HPLC analysis.

### RNA isolation and quantitative real-time expression analysis

Liquid nitrogen was used to grind the hairy roots (three samples of control and each treatment), which were then lysed in Trizol reagent (Thermo Fisher Scientific, USA). The RNA was subsequently extracted following the manufacturer’s instructions. The concentration and purity of the RNA were measured using a NanoDrop™ 1000 Spectrophotometer (Thermo Fisher Scientific, USA). A DNase I, RNase-free kit (Thermo Fisher Scientific, USA) was used to remove any DNA contamination. DNase-treated RNA (1000ng) was reverse-transcribed into first-strand cDNA using TOPscript™ RT DryMIX (dT18) (Enzynomics, South Korea). Previously published primers for the studied genes^8^ were obtained from Macrogen (South Korea). The primers used were as follows: tryptophan decarboxylase gene (TDC), Forward: TCCGAAAACAAGCCCATCGT, Reverse: AAGGAGCGGTTTCGGGGATA; strictosidine synthase gene (STR), Forward: TGACAGTCCCGAAGGTGTGG, Reverse: CGCCGGGAACATGTAGCTCT; Ribosomal Protein S9 (RPS9), forward: TCCACCATGCCAGAGTGCTCATTA, Reverse: TCCATCACCACCAGATGCCTTCTT. Rps9 was used as the housekeeping gene. In a 20-µL RT-qPCR reaction volume, 1 µL of cDNA, 1 µL of forward and reverse primers (10 µM each), 10 µL of TOPreal™qPCR 2× PreMIX (SYBR Green with low ROX) from Enzynomics in South Korea, and DNase-free water were used. The amplification process was performed in triplicate for each sample. It began with a 15-minute denaturation step at 95 degrees Celsius, followed by 40 cycles. Each cycle consisted 10 s at 95 degrees Celsius, 15 s at 60 degrees Celsius, and 30 s at 72 degrees Celsius. After each reaction, a melting curve analysis was conducted to ensure the specificity of the amplicons produced. The Ct values obtained from the real-time PCR machine were then transferred to Microsoft Excel for analysis using the 2^−ΔΔ Ct^ method, as described by Livak & Schmittgen in 2001^19^.

### Statistical analysis

Statistical analysis was performed using one-way ANOVA, followed by Duncan’s multiple comparison test. The Statistical Package for Social Sciences (SPSS version 16.0, SPSS Inc., Chicago, IL, USA) was utilized for this analysis. All observed variations were found to be statistically significant with a p-value of 0.05 or less.

## Results and discussions

**Fig. 3:**
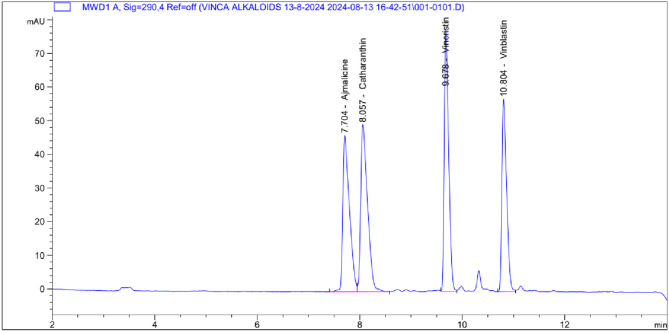
HPLC chromatogram of ajmalicine, catharanthine, vincristine, and vinblastine.

In this study, our goal is to improve the production of valuable indole alkaloids (ajmalicine, catharanthine, vincristine, and vinblastine) in the hairy root culture of *C. roseus* by employing various strategies. These strategies include using biotic and abiotic elicitors (yeast extract and methyl jasmonate) as well as feeding precursors (tryptophan and tryptamine). Calibration curves (Fig. 3) were created for ajmalicine, catharanthine, vincristine, and vinblastine by plotting the peak area from the chromatograms against different concentrations of each substance. All metabolite levels are reported in µg/g dry weight (DW) for consistency.

**Fig. 4:**
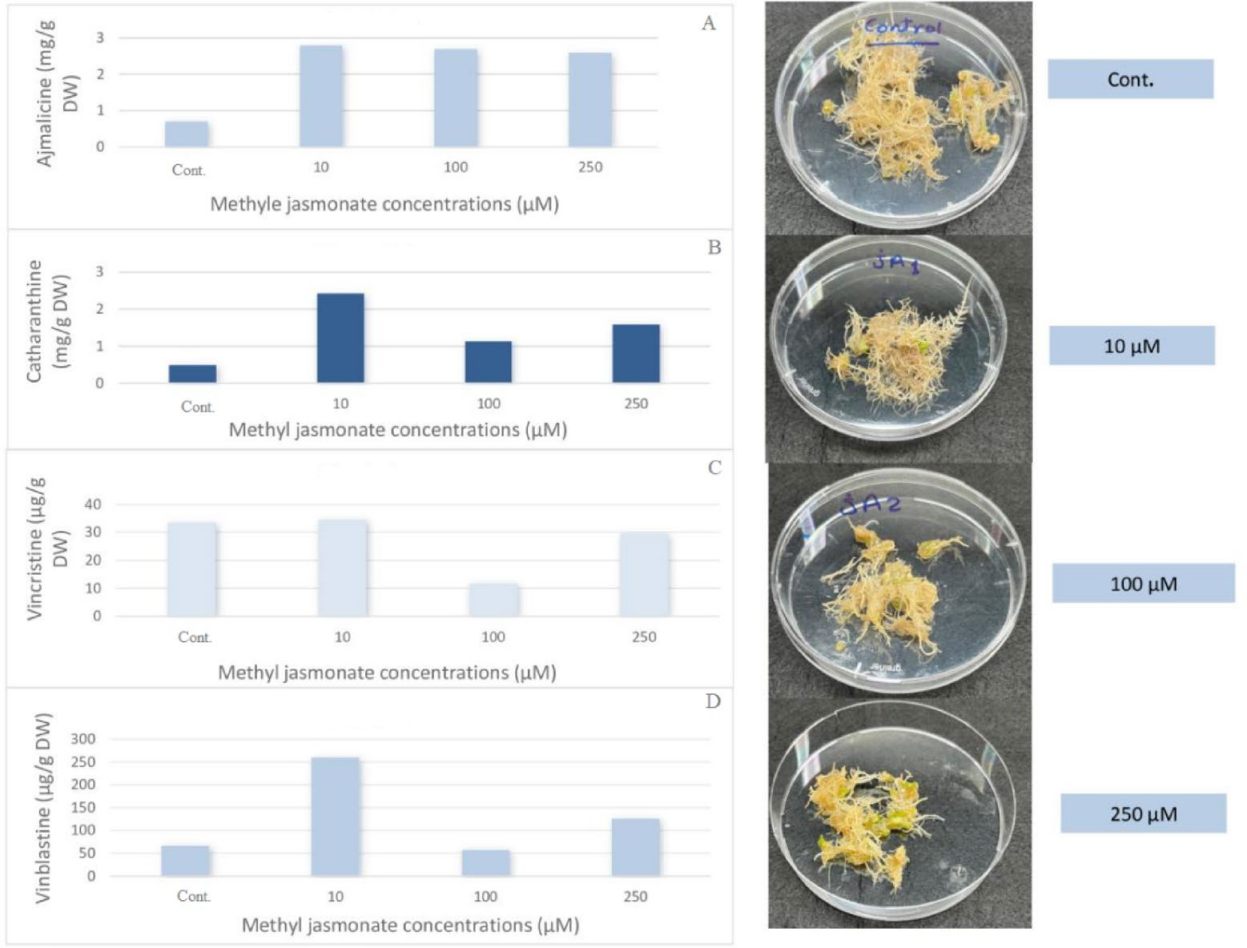
Effect of different methyl jasmonate concentrations on the accumulation of (**A**) ajmalicine, (**B**) catharanthine, (**C**) vincristine, and (**D**) vinblastine n *C. roseus* hairy root culture after one week.

The impact of varying concentrations of methyl jasmonate (10, 100, and 250 µM) as an abiotic elicitor on the accumulation of valuable indole alkaloids in *C. roseus* hairy root cultures is illustrated in Fig. 4. The results indicate that all concentrations of methyl jasmonate increased the levels of ajmalicine and catharanthine. However, when it came to vincristine and vinblastine, some concentrations increased the levels while others decreased them. The concentration of 10 µM was the most effective for all compounds of indole alkaloids, yielding 2.789 mg/g DW of ajmalicine, 2.420 mg/g DW of catharanthine, 34.54 µg/g DW of vincristine, and 260 µg/g DW of vinblastine compared to the control, which yielded 0.697 mg/g DW of ajmalicine, 0.486 mg/g DW of catharanthine, 34.54 µg/g DW of vincristine, and 66.53 µg/g DW of vinblastine.

Methyl jasmonate is known to activate jasmonate signaling, leading to the upregulation of transcription factors (e.g., Octadecanoid-Derivative Responsive Catharanthus AP2-domain proteins (ORCAs)) that bind to promoter regions of TIA pathway genes, thereby enhancing alkaloid biosynthesis (Paul et al., 2017). Mona et al.^20^ previously observed that adding 100 µM of methyl jasmonate to the cell suspension of *C. roseus* resulted in a 19-fold increase in ajmalicine compared to the control. Akhgari et al.^21^ found that, when compared to control cultures, all three concentrations of MeJA (50 µM, 100 µM, and 200 µM) enhanced the accumulation of indole alkaloids in hairy root cultures of *Rhazya stricta* at four different exposure times (1, 3, 5, and 7 days). Karakaş^22^ increased the production of indole alkaloids in the in vitro plantlets of *Isatis demiriziana Mısırdalı* by applying varying concentrations of methyl jasmonate in the solid Murashige-Skoog (MS) medium. He found that 1.0 mM MeJA had the largest increase in tryptanthrin synthesis, roughly 2.85 times greater than the control.

**Fig. 5:**
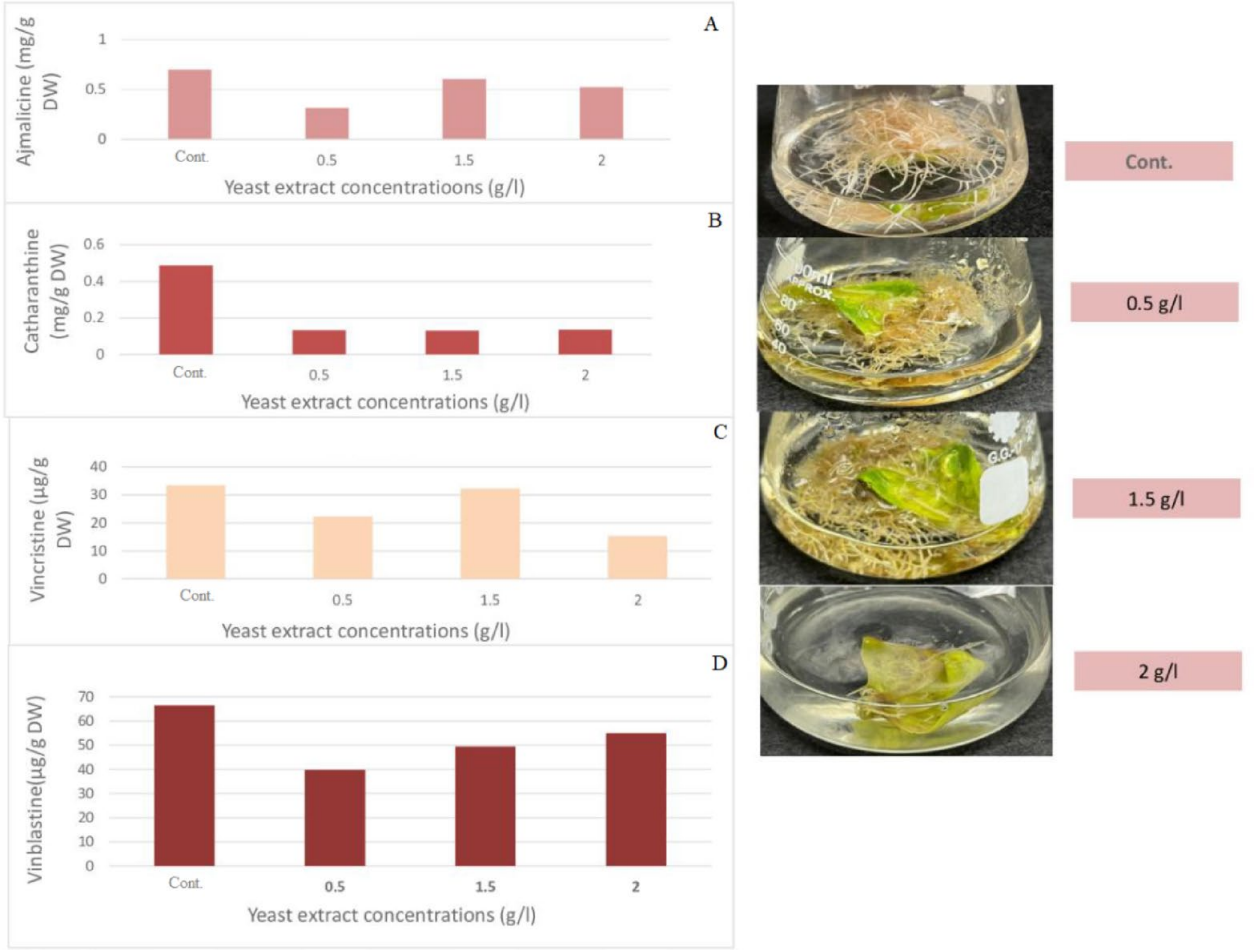
Effect of different yeast extract concentrations on the accumulation of of (**A**) ajmalicine, (**B**) catharanthine, (**C**) vincristine, and (**D**) vinblastine in *C. roseus* hairy root culture after one week.

Various concentrations of yeast extract as a biotic elicitor (0.5, 1.5, 2.0 g/l) on the accumulation of different indole alkaloids in *C. roseus* hairy root culture were presented in Fig. 5. The results showed that treatments with different concentrations of yeast extract decreased all indole alkaloids compared to the control. Only 1.5 g/l of yeast extract accumulated vincristine (32.26 µg/g DW) to a level approximately equal to the control (33.46 µg/g DW). Further concentrations are required to confirm that yeast extract inhibits indole alkaloid accumulation. It is also recommended to explore lower and higher exposure periods.

Root biomass (fresh and dry weight) did not significantly decrease at the tested yeast extract concentrations, suggesting the reduction in alkaloids was not due to toxicity. Possible reasons include nutrient imbalance leading to redirected metabolism, stress-induced growth inhibition, or potential feedback inhibition at high concentrations.

Maqsood & Abdul^16^ used yeast extract to enhance vinblastine and vincristine in plantlets and tissues produced from protoplasts in *C. roseus*. The addition of yeast extract to the media increased the yield of vinblastine and vincristine in growing tissues, with the highest levels observed in in vitro-grown leaves and germinating embryos. The maximum yield, 22.74% vinblastine and 48.49% vincristine enrichment in germinating embryos, was achieved with the treatment of 1.5 mg/l yeast extract. Khashan & Husain^23^ increased vinblastine and vincristine in a callus culture of *C. roseus* by using yeast extract as a biotic elicitor. According to their research, the greatest amount was observed with the 2 g/L yeast extract treatment, which significantly raised vinblastine and vincristine levels compared to the control. Hegazi et al.^24^ utilized yeast extract from *Ephedra alata* L. cell suspension cultures to enhance the production of alkaloids, particularly ephedrine. After 24 days of exposure, the maximum ephedrine accumulation was observed at a 15% concentration of yeast extract, resulting in a value of 2.375% in terms of 2.589 mg of total dry weight.

**Fig. 6:**
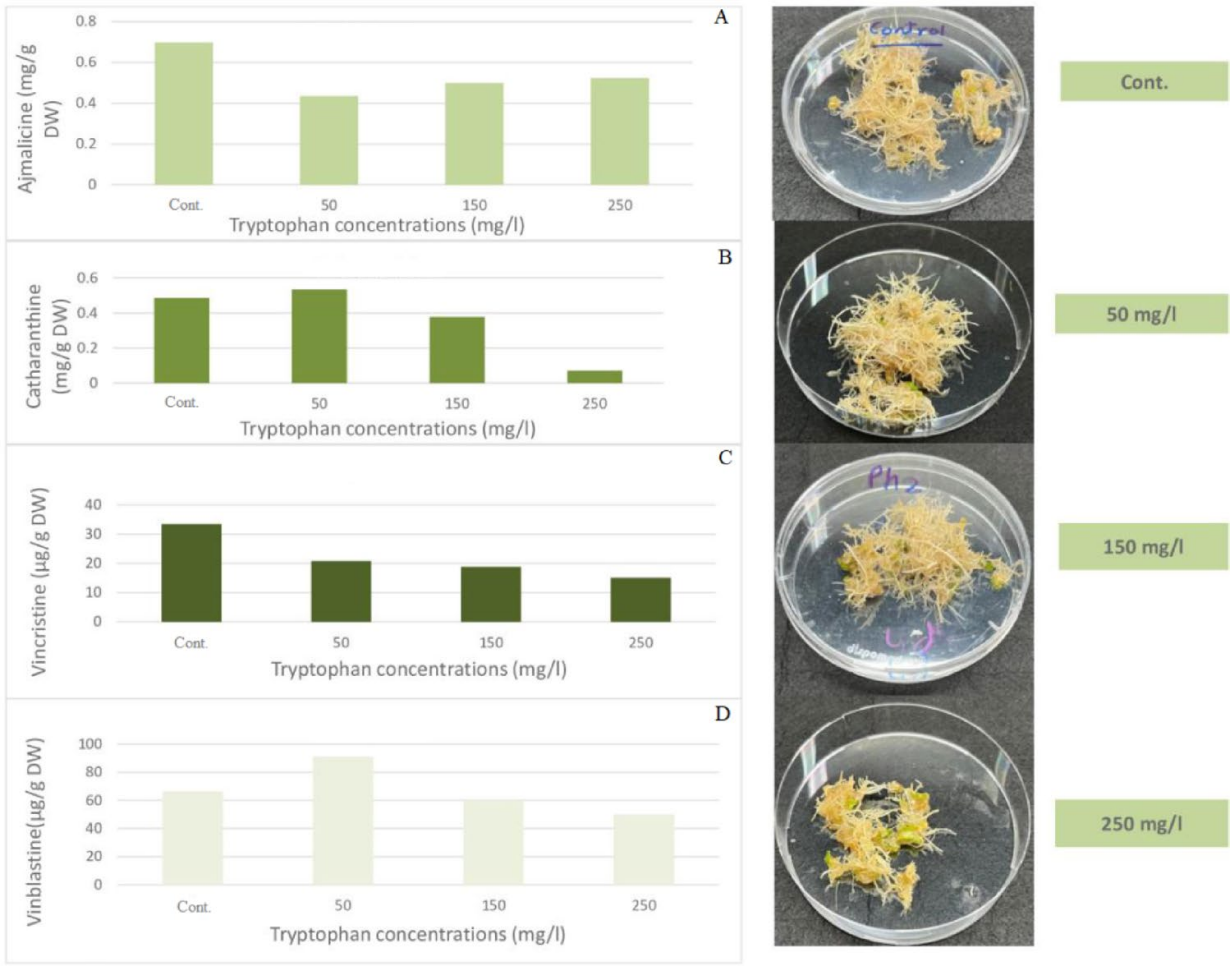
Effect of different tryptophan concentrations on the accumulation of (**A**) ajmalicine, (**B**) catharanthine, (**C**) vincristine, and (D) vinblastine in *C. roseus* hairy root culture after one week.

Different concentrations of tryptophan as a precursor (50, 150, 250 mg/L) were tested to enhance indole alkaloid production in *C. roseus* hairy root culture. The results in Fig. 6 show that a concentration of 50 mg/L of tryptophan had a positive effect on the accumulation of both catharanthine and vinblastine levels, achieving 0.533 mg/g DW and 91.14 µg/g DW, respectively. This was compared to the control, which achieved 0.486 mg/g DW and 66.53 µg/g DW, respectively. The other concentrations decreased the levels of both indole alkaloids (catharanthine and vinblastine). However, ajmalicine and vincristine decreased in all concentrations of tryptophan used. Tryptophan acts as a direct precursor for terpenoid indole alkaloid (TIA) biosynthesis in *Catharanthus roseus*, significantly enhancing the production of valuable metabolites like catharanthine (up to 50.96 µg/g DW) and ajmalicine (up to 2.686 µg/g DW). It also boosts catharanthine, vinblastine, and vincristine levels via metabolic pathway regulation^13^. Cessur et al.^25^ previously used tryptophan as a precursor to enhance indole alkaloids in root cultures of *Isatis tinctoria* L. Indole alkaloids were elevated at 200 and 300 mg/L compared to the control. The roots treated with 200 mg/L had the highest concentration.

In *C. roseus* callus cultures, Jassim & Ameen^26^ found that the maximum accumulation of vincristine occurred at 200 mg/L of tryptophan, followed by 300 and 400 mg/L. This supports the idea that tryptophan has a dose-dependent effect on alkaloid accumulation. Tryptophan was also shown to be an efficient precursor that enhances the formation of alkaloids when added to in vitro cultures at the appropriate concentration.

In a study conducted by Krishnan et al.^27^, it was discovered that the use of 1 mM and 2 mM Tryptophan resulted in an increase in the production of the quinoline alkaloid camptothecin in cell cultures of Nothapodytes nimmoniana. The impact of specific precursors such as L-phenylalanine and L-tyrosine, as well as elicitors like chitosan and methyl jasmonate, on *C. roseus* was examined to enhance the levels of vincristine and vinblastine. The growth of TUGAS hairy roots was investigated to improve the production of vincristine and vinblastine, as reported by Phuong Thi Bach Vu and colleagues^9^. The results indicated that hairy roots have the potential to produce both vincristine and vinblastine when provided with the appropriate starting materials.

**Fig. 7:**
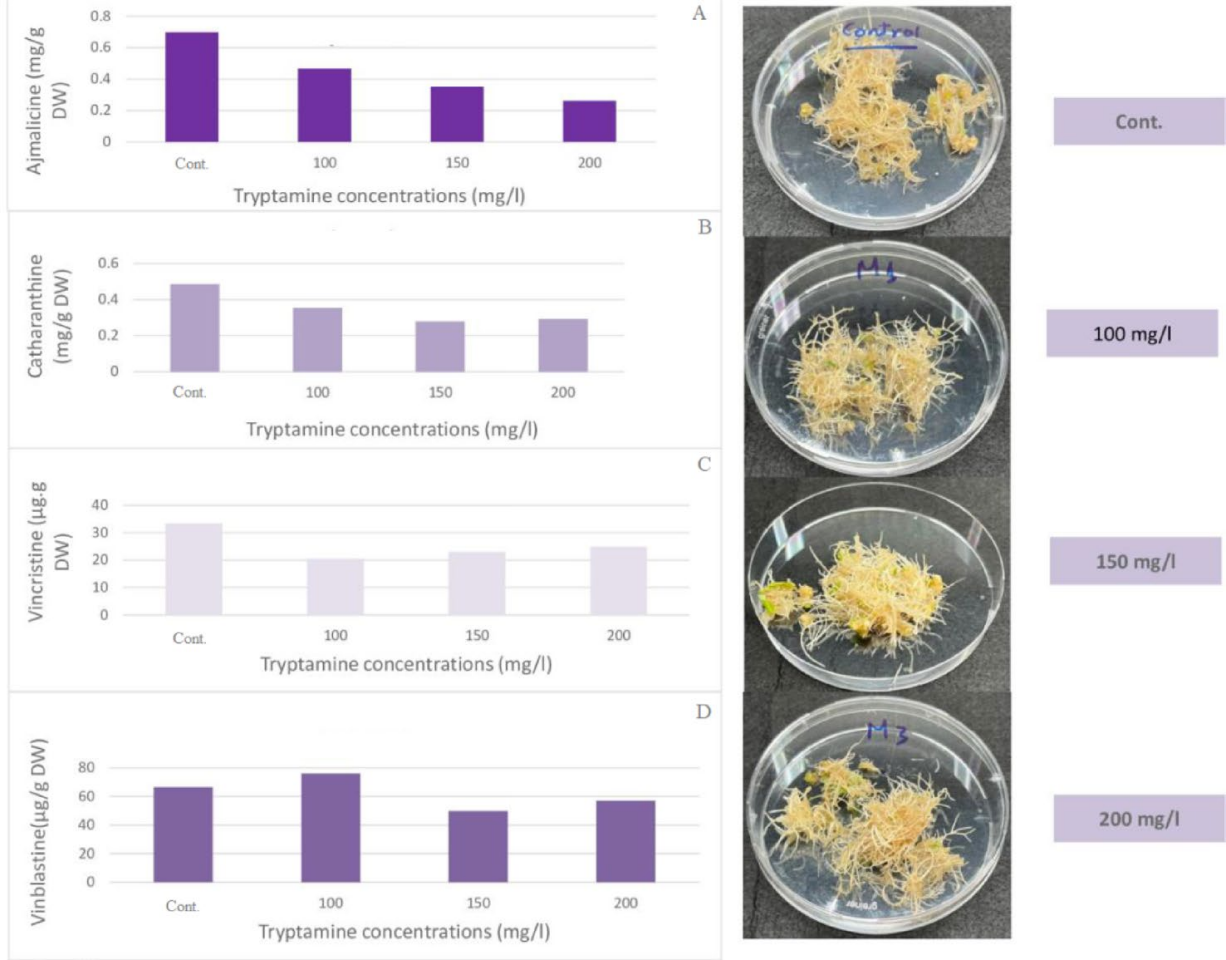
Effect of different tryptamine concentrations on the accumulation of (**A**) ajmalicine, (**B**) catharanthine, (**C**) vincristine, and (Dalia) vinblastine in *C. roseus* hairy root culture after one week.

Several concentrations of tryptamine (100, 150, and 200 mg/l) were investigated as a precursor to increase the formation of indole alkaloids in *C. roseus* hairy root culture. The data in Fig. 7 shows that only vinblastine was enhanced when using 100 mg/l of tryptamine, resulting in 75.99 µg/g DW compared to the control, which was 66.53 µg/g DW. On the other hand, other indole alkaloids (ajmalicine, catharanthine, and vincristine) decreased with all concentrations of tryptamine used.

According to Whitmer et al.^28^, alkaloid accumulation was further increased when tryptamine was given in addition to the iridoid precursors. Sharma et al.^14^ reported that the highest alkaloid levels, reaching 6.2% of dry weight, were found in shoot cultures of *C. roseus* fed with 1000 mg/l of tryptamine. The initial substrate for hundreds of monoterpene indole alkaloids is tryptamine^2^. Tryptophan decarboxylase produces this substrate enzymatically from tryptophan^29^.

### Gene expression studies

**Fig. 8:**
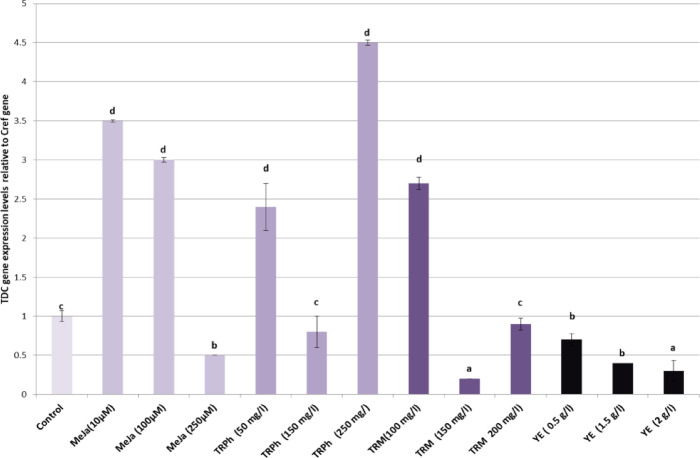
The mRNA expression levels for the *TDC* gene were measured in the control plants as well as in plants treated with different doses of Methyl jasmonate, Tryptophan, Tryptamine or Yeast extract. The bars represent the standard error from the mean. Mean values with different superscript letters indicate significant differences (*p* ≤ 0.05).

**Fig. 9:**
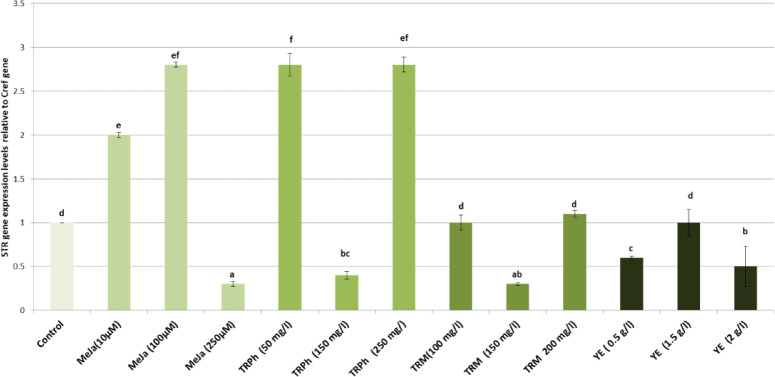
The mRNA expression levels for the *STR* gene were measured in the control plants as well as in plants treated with different doses of Methyl jasmonate, Tryptophan, Tryptamine or Yeast extract. The bars represent the standard error from the mean. Mean values with different superscript letters indicate significant differences (*p* ≤ 0.05).

In the present study, alterations in the gene expression of *STR* and *TDC* genes in hairy roots of *C. roseus* were evaluated using qPCR in response to different concentrations of Methyl Jasmonate (MeJa), tryptophan (TRPh), tryptamine (TRM), or yeast extract (YE) as shown in Figs. 8 and 9. The hairy roots treated with both doses of methyl jasmonate (10 and 100 µM) exhibited a significant increase in the expression of *STR* (2 and 2.8 fold; *p* < 0.05) and *TDC* genes (3.5 and 3 fold; *p* < 0.05), respectively compared to the control treatment. However, the high dose (250 µM) significantly reduced the expression levels of both genes. Similarly, doses of 50 and 250 mg/L of tryptophan induced significantly higher expression levels of *STR* (2.8 and 2.8 fold; *p* < 0.05) and *TDC* genes (2.4 and 4.5 fold; *p* < 0.05) compared to the control. However, the dose of 150 mg/L in the medium significantly decreased the expression levels of both genes. Tryptophan serves as a direct precursor for tryptamine via TDC. Increased substrate availability likely drives flux into the TIA pathway, leading to higher accumulation of downstream alkaloids under optimal concentrations^30^. The expression levels of the *TDC* gene increased significantly after treatment with 100 mg/L of Tryptamine (2.7 fold; *p* < 0.05). Meanwhile, *STR* expression levels remained unchanged. A dose of 150 mg/L of Tryptamine decreased both *STR* and *TDC* expression, while a dose of 200 mg/L resulted in equal values for *STR* and *TDC* expression compared to the control. Treatment with yeast extract at different concentrations in the medium significantly reduced the expression levels of *TDC* compared to the control. Additionally, yeast extract at concentrations of 0.5 and 2.0 g/L significantly decreased the expression levels of *STR*, whereas a dose of 1.5 g/L showed no significant change compared to the control. yeast extract (YE) acts as a biotic elicitor that generally induces, rather than suppresses, the expression of TDC and STR genes *in Catharanthus roseus* cell suspensions, triggering terpenoid indole alkaloid (TIA) biosynthesis. While YE induce these genes, it also induces repressor proteins zinc finger proteins (ZCT1, 2, 3) that can repress gene expression via binding to their promoters^31^. It should be noted that gene expression peaks may occur earlier (e.g., 24–48 h), and future studies will include earlier time points to capture transcriptional dynamics.

Several authors have studied the activity levels of the *TDC* and *STR* genes in response to elicitors. Overexpression of these genes may lead to elevated levels of alkaloid production, including vindoline, vinblastine, and catharanthine^13^. The gene expression analysis of the elicited multiple shoot cultures of *C. Roseus* showed a very slight increase in the levels of the studied genes^14^.

In our previous study, it was found that the two elicitors, methyl jasmonate (MeJa) and UV-B irradiation induce the tryptophan decarboxylase gene expression differently. Additionally, their combined application induces the tryptophan decarboxylase gene but to a lesser extent for the strictosidine synthase gene expression in the treated *C. roseus* leaves^8^. The study investigated how different light intensities affect the expression levels of two key genes, *CrTDC* and *CrSTR*, in the leaves of C. Roseus plants from different genotypes, as noted in Gholizadeh et al.^10^. It was discovered that plants grown in low-light conditions exhibited significantly higher levels of *CrSTR* expression in their leaves compared to those grown under normal light. However, there was no notable difference in the expression of the *CrTDC* gene. Shading did not significantly affect the CrTDC expression level. In contrast, the expression of the *CrSTR* gene was upregulated in shaded plants.

Possible mechanisms for dimeric alkaloid detection in hairy roots: Although hairy roots are not typically known to produce vindoline-dependent dimers like vinblastine and vincristine, our detection of corresponding peaks under specific elicitation conditions suggests potential pathway activation, cross-talk, or the presence of trace leaf-like cells. These findings warrant further validation using LC-MS/MS.

## Conclusion

In conclusion, the current results demonstrate how elicitors affect the accumulation of anticancer compounds, and consequently, gene expression levels in *C. roseus* hairy roots. Our results indicate that a concentration of 10 µM was found to be the most effective for all compounds of indole alkaloids. Addtionally, methyl jasmonate (10 and 100 µM), tryptophan (50 and 250 mg/l), and tryptamine (100 mg/l) significantly increased the expression of *STR* and *TDC* genes compared to the control treatment. In contrast to other elicitors, treatment with yeast extract at different concentrations significantly decreased all indole alkaloids and also reduced the expression levels of *STR* and *TDC* compared to the control. Future work will focus on scaling up the most effective treatments (e.g., 10 µM MeJA and 50 mg/L tryptophan) in bioreactor systems to assess their industrial applicability and consistency. For future studies, it is recommended to conduct a time-course analysis (e.g., 1, 3, 7, 14 days) to determine the optimal accumulation windows. Additionally, validation of alkaloid identity using LC-MS/MS is advised. Including earlier time points to capture transcriptional dynamics is also recommended for further study. A limitation of the current study is pooling three samples per treatment for a single, non-repeated HPLC analysis.

## Data Availability

The datasets generated and/or analyzed during the current study are available from the corresponding author on reasonable request.

## References

[1] Verma, A. K., Singh, R. R. & Singh, S. Improved alkaloid content in callus culture of C. roseus. *Bot. Serbica*. **36** (2), 123–130 (2012). https://api.semanticscholar.org/CorpusID:39737628

[2] van Der Heijden, R., Jacobs, D. I., Snoeijer, W., Hallard, D. & Verpoorte, R. The C. alkaloids: pharmacognosy and biotechnology. *Curr. Med. Chem.* **11** (5), 607–628. 10.2174/0929867043455846 (2004). 15032608 10.2174/0929867043455846

[3] Kumar, S., Singh, B. & Singh, R. C. Roseus (L.) G. Don: A review of its ethnobotany, phytochemistry, ethnopharmacology and toxicities. *J. Ethnopharmacol.* **284** (4), 114647. 10.1016/j.jep.2021.114647 (2022). 34562562 10.1016/j.jep.2021.114647

[4] Wang, Q. et al. Development of efficient *C. roseus* regeneration and transformation system using Agrobacterium tumefaciens and hypocotyls as explants. *BMC Biotechnol.* **12** (1), 34. 10.1186/1472-6750-12-34 (2012). 22748182 10.1186/1472-6750-12-34 PMC3483169

[5] Hanafy, M. S., Matter, M. A., Asker, M. S. & Rady, M. R. Production of Indole alkaloids in hairy root cultures of C. roseus L. and their antimicrobial activity. *S Afr. J. Bot.* **105**, 9–18. 10.1016/j.sajb.2016.01.004 (2016).

[6] Pan, Q., Saiman, M., Zuwairi, Verpoorte, R. & Tang, K. Accumulation of terpenoid Indole alkaloids in jasmonic acid elicited C. roseus plants before and during flowering. *Pak. J. Bot.* **50** (3), 1077–1083 (2018).

[7] Baenas, N., García-Viguera, C. & Moreno, D. A. Elicitation: a tool for enriching the bioactive composition of foods. *Molecules* **19** (9), 13541–13563. 10.3390/molecules190913541 (2014). 25255755 10.3390/molecules190913541 PMC6270998

[8] Rady, M. R., Gierczik, K., Ibrahem, M. M., Matter, M. A. & Galiba, G. Anticancer compounds production in C. roseus by Methyl jasmonate and UV-B elicitation. *S Afr. J. Bot.* **142**, 34–41. 10.1016/j.sajb.2021.05.024 (2021).

[9] Vu, P. T. B. et al. In vitro growth and content of vincristine and vinblastine of C. roseus L. hairy roots in response to precursors and elicitors. *Plant. Sci. Today*. **9** (1), 21–28. 10.14719/pst.1337 (2022).

[10] Gholizadeh, F. et al. Growth light substantially affects both primary and secondary metabolic processes in C. roseus plants. *Photosynthetica* **61** (4), 451–460. 10.32615/ps.2023.037 (2023). 39649484 10.32615/ps.2023.037 PMC11586840

[11] Rady, M. R., Amer, A. M., Ibrahim, M. M., Elminisy, A. & Bekheet, S. Different carbon sources and their concentrations affect alkaloid accumulation in transformed root cultures of C. roseus. *Egypt. J. Chem.* **68** (2), 211–219. 10.21608/ejchem.2024.292105.9759 (2025).

[12] Pasquali, G. et al. Coordinated regulation of two Indole alkaloid biosynthetic genes from C. roseus by auxin and elicitors. *Plant. Mol. Biol.* **18** (6), 1121–1131. 10.1007/bf00047715 (1992). 1600148 10.1007/BF00047715

[13] Sharma, A., Verma, P., Mathur, A. & Mathur, A. K. Overexpression of Tryptophan decarboxylase and Strictosidine synthase enhanced terpenoid Indole alkaloid pathway activity and antineoplastic vinblastine biosynthesis in C. roseus. *Protoplasma* **255** (5), 1281–1294. 10.1007/s00709-018-1233-1 (2018). 29508069 10.1007/s00709-018-1233-1

[14] Sharma, A., Mathur, A. K., Ganpathy, J., Joshi, B. & Patel, P. Effect of abiotic elicitation and pathway precursors feeding over terpenoid Indole alkaloids production in multiple shoot and callus cultures of C. roseus. *Biol. (Bratisl)*. **74** (5), 543–553. 10.2478/s11756-019-00202-5 (2019).

[15] Ruiz-May, E., Galaz-Avalos, R. M. & Loyola-Vargas, V. M. Differential secretion and accumulation of terpene indole alkaloids in hairy roots of Catharanthus roseus treated with methyl jasmonate. * Mol. Biotechnol.* **41**(3), 278–285 (2009). 18841500 10.1007/s12033-008-9111-2

[16] Maqsood, M. & Abdul, M. Yeast extract elicitation increases vinblastine and vincristine yield in protoplast derived tissues and plantlets in C. roseus. *Rev. Bras. Farmacogn*. **27** (5), 549–556. 10.1016/j.bjp.2017.05.008 (2017).

[17] Pandiangan, D., Tilaar, W., Nainggolan, N. & Wahyud, L. Relations between catharanthine content enhancement with the other associated secondary metabolites in catharanthus roseus cell culture that treated Tryptophan. *Int. J. Sci. Res.* ;6–14. (2013).

[18] Rahmatzadeh, S., Khara, J. & Kazemitabar, S. K. The study of in vitro regeneration and growth parameters in catharanthus roseus L. under application of Tryptophan. *J. Sci. Kharazmi Univ.* **14** (3), 249–260 (2014). http://jsci.khu.ac.ir/article-1-1495-en.html

[19] Livak, K. J. & Schmittgen, T. D. Analysis of relative gene expression data using realtime quantitative PCR and 2(-Delta delta C(T)) method. *Methods* **25** (4), 402–408. 10.1006/meth.2001.1262 (2001). 11846609 10.1006/meth.2001.1262

[20] Ibrahim, M. M., Danial, N., Matter, M. A. & Rady, M. R. Effect of light and Methyl jasmonate on the accumulation of anticancer compounds in cell suspension cultures of C. roseus. *Egypt. Pharm. J.* **20** (4), 294–302. 10.4103/epj.epj_48_21 (2021).

[21] Akhgari, A. et al. Methyljasmonate elicitation increases terpenoid Indole alkaloid accumulation in rhazya stricta hairy root cultures. *Plants* **8** (12), 534. 10.3390/plants8120534 (2019). 31766620 10.3390/plants8120534 PMC6963348

[22] Karakaş, Ö. Effects of Methyl jasmonate and Putrescine on tryptanthrin and indirubin production in in vitro cultures of isatis Demiriziana Mısırdalı. *Int. J. Second Metab.* **6** (3), 241–250. 10.21448/ijsm.521498 (2019).

[23] Khashan, K. T. & Husain, M. A. Effect of biotic factors stresses on vinblastine and vincristine production from callus of C. roseus. *Euphrates J. Agric. Sci.* **7** (2), 25–41 (2015).

[24] Hegazi, G., Ghareb, H. & Gabr, M. Ephedrine production from suspension cultures of ephedra Alata L. callus. *BioTechnologia* **101** (1), 25–33. 10.5114/bta.2020.92925 (2020).

[25] Cessur, A., Tuğlu, Ü., Albayrak, İ & Baydar, N. G. Molecular and biochemical insights into tryptophan-induced indole alkaloid biosynthesis in Isatis tinctoria L. root cultures. *Plant. Cell. Tissue Organ. Cult.*10.1007/s11240-025-03106-2 (2025).

[26] Jassim, E. H. & Ameen, S. K. Influence of L-Trp and Salicylic acid on secondary metabolites production from leaves induced cal¬lus of C. roseus LG don in vitro. *J. Biotech. Res. Cent.* **8** (2), 35–43. 10.24126/jobrc.2014.8.2.326 (2014).

[27] Krishnan, N. et al. Effect of abiotic elicitor on the production of camptothecin from nothapodytes nimmoniana (J. Graham) Mabb. *Biocatal. Agric. Biotechnol.* **53** (1), 102846. 10.1016/j.bcab.2023.102846 (2023).

[28] Whitmer, S., Van Der Heijden, R. & Verpoorte, R. Effect of precursor feeding on alkaloid accumulation by a strictosidine synthase over-expressing transgenic cell line S1 of C. roseus.. *In Plant Cell Tissue and Organ Culture* **69**(1), 85–93 (2002). 10.1016/s0168-1656(02)00027-5 12039535

[29] Facchini, P. J., Huber-Allanach, K. L. & Tari, L. W. Plant aromatic L-amino acid decarboxylases: evolution, biochemistry, regulation, and metabolic engineering applications. *Phytochemistry* **54** (2), 121–138. 10.1016/s0031-9422(00)00050-9 (2000). 10872203 10.1016/s0031-9422(00)00050-9

[30] Yousefi, F. et al. Evaluation of the expression pattern of TIAs pathway genes in response to Tryptophan amino acid treatment and drought stress in catharanthus roseus. *PLoS One*. **20** (10), e0333313. 10.1371/journal.pone.0333313 (2025). 41066403 10.1371/journal.pone.0333313 PMC12510576

[31] Paul, B. et al. Zinc finger proteins act as transcriptional repressors of alkaloid biosynthesis genes in catharanthus roseus. *J. Biol. Chem.* **279** (51), 52940–52948. 10.1074/jbc.M404391200 (2004). 15465826 10.1074/jbc.M404391200

