# Correction: Enhanced accumulation of anticancer compounds in *C. roseus* hairy root cultures through elicitation and precursor feeding

**DOI:** 10.1038/s41598-026-63850-z

**Published:** 2026-08-05

**Authors:** Mohamed R. Rady, Dalia M. Mabrouk, Alaa M. El-Minisy, Mona M. Ibrahim

**Affiliations:** 1 Department of Plant Biotechnology, Biotechnology Research Institute, National Research Centre, Cairo, 12622 Egypt; 2 Department of Cell Biology, Biotechnology Research Institute, National Research Centre, Cairo, 12622 Egypt

Correction to: *Scientific Reports* 10.1038/s41598-026-36185-y, published online 03 February 2026

In the original version of the article, author Alaa M. El-Minisy was omitted from the author list.

The Author Contributions section now reads:

MRR designed the research project, contributed to writing the manuscript and revised the manuscript. MMI performed sample preparation, HPLC analysis, and participated in writing the manuscript. DMM conducted RNA isolation, cDNA synthesis, qPCR, data analysis, and contributed to writing manuscript. AME induced hairy root and culture maintenance to suspension stage, image documentation.

The original Article has been corrected.

